# Modeling undetected poliovirus circulation following the 2022 outbreak in the United States

**DOI:** 10.1080/14760584.2023.2299401

**Published:** 2024-01-04

**Authors:** Dominika A. Kalkowska, Kamran Badizadegan, Janell A. Routh, Cara C. Burns, Eli S. Rosenberg, I. Ravi Brenner, Jane R. Zucker, Marisa Langdon-Embry, Kimberly M. Thompson

**Affiliations:** a Kid Risk, Inc, Orlando, FL, USA;; b Division of Viral Diseases, Centers for Disease Control and Prevention, Atlanta, GA, USA;; c Office of Public Health, New York State Department of Health, Albany, NY, USA;; d Department of Epidemiology and Biostatistics, State University of New York at Albany, Albany, NY, USA;; e New York City Department of Health and Mental Hygiene, New York, NY, USA;; f Immunization Services Division, Centers for Disease Control and Prevention, Atlanta, GA, USA;; g Department of Public Health, Syracuse University, Syracuse, NY, USA

**Keywords:** Polio, eradication, dynamic modeling, outbreak response, IPV

## Abstract

**Background:**

New York State (NYS) reported a polio case (June 2022) and outbreak of imported type 2 circulating vaccine-derived poliovirus (cVDPV2) (last positive wastewater detection in February 2023), for which uncertainty remains about potential ongoing undetected transmission.

**Research Design and Methods:**

Extending a prior deterministic model, we apply an established stochastic modeling approach to characterize the confidence about no circulation (CNC) of cVDPV2 as a function of time since the last detected signal of transmission (i.e. poliovirus positive acute flaccid myelitis case or wastewater sample).

**Results:**

With the surveillance coverage for the NYS population majority and its focus on outbreak counties, modeling suggests a high CNC (95%) within 3–10 months of the last positive surveillance signal, depending on surveillance sensitivity and population mixing patterns. Uncertainty about surveillance sensitivity implies longer durations required to achieve higher CNC.

**Conclusions:**

In populations that maintain high overall immunization coverage with inactivated poliovirus vaccine (IPV), rare polio cases may occur in un(der)-vaccinated individuals. Modeling demonstrates the unlikeliness of type 2 outbreaks reestablishing endemic transmission or resulting in large absolute numbers of paralytic cases. Achieving and maintaining high immunization coverage with IPV remains the most effective measure to prevent outbreaks and shorten the duration of imported poliovirus transmission.

## 1. Introduction

In 2022, the United States (US) reported local transmission of an imported type 2 circulating vaccine-derived poliovirus (cVDPV2) in an under-vaccinated community in New York State (NYS). This followed the detection of a clinical case of paralytic poliomyelitis (polio) in a young adult detected by acute flaccid myelitis (AFM) surveillance with paralysis onset in June 2022 [1–3]. This experience demonstrated the potential for sustained transmission of poliovirus in communities with relatively low immunization coverage in the US, despite high reported overall national coverage of 93.1% for kindergartners in the 2022–2023 school year with recommended doses of inactivated poliovirus vaccine (IPV) [4]. Recent modeling of the 2022 New York polio outbreak [3] shows routine immunization coverage trends for NYS overall and the decrease during and since the COVID-19 pandemic (Supplement Figure S1 in [3]), as well as heterogeneity in vaccine coverage by county that highlights the concentration of un(der) vaccination in the outbreak counties (Figure 1C in [3]).

Detection of circulating poliovirus in an under-vaccinated population in NYS coincided with similar reports from socially connected communities in the United Kingdom (UK) and Israel. Contemporaneous analyses of genetic sequences from wastewater samples collected in the US, the UK, and Israel identified linkage between the outbreak polioviruses in all three countries [2,5,6]. Canada subsequently reported detecting genetically linked viruses in a limited wastewater surveillance activity without evidence of local transmission [7]. Similar to the US, Israel reported a polio case (paralysis onset in February 2023 [8]), but the UK did not detect paralytic cases resulting from its local cVDPV2 transmission.

Poliovirus transmission in populations that use IPV with high overall immunization coverage can occur in the absence of detectable paralytic cases [9,10]. Since IPV protects against paralysis (but not infection) for vaccine recipients, high IPV coverage implies very low chances of paralytic poliomyelitis despite ongoing poliovirus infections, due to the relative scarcity of fully susceptible individuals. Paralysis only occurs in a relatively small fraction of fully susceptible individuals, and only clinical presentations that meet strict criteria for paralytic poliomyelitis count as confirmed polio cases [11]. As such, (re)infection of immunologically naive and immune individuals can occur without causing paralysis, such that all individuals in the population may participate in transmission (albeit with different levels of participation), resulting in undetected poliovirus transmission in the community. Broad environmental surveillance programs may help detect poliovirus transmission in clinically silent communities, but such programs pose other significant challenges [12].

Once poliovirus transmission has been established in a community, die-out of transmission may occur stochastically and depends on the epidemiological conditions and immunization status of the population [13–15]. Numerous prior studies explored the potential for undetected transmission of polioviruses and the importance of high-quality surveillance information for confirmation of die-out [16–21]. Environmental surveillance systems (including wastewater surveillance) for polioviruses vary substantially in design and continue to evolve, with variable sensitivities of the systems that depend on the population, frequency, amount, and nature of the samples collected, and specific laboratory assays used [22]. The absence of positive wastewater detections in NYS since February 2023 raises questions about whether the transmission of the outbreak virus in NYS has ended [3]. To address this question, we apply stochastic modeling and analysis of surveillance information to assess confidence about no circulation in NYS and the probability of die-out for this outbreak as a function of time since the last detected signal.

## 2. Methods

We extended a prior deterministic model of the NYS outbreak that suggested potential die-out of the transmission and identified the need for further stochastic modeling to explore the die-out dynamics [3]. Briefly, the model distributed the NYS population between outbreak counties (OC) and non-outbreak counties (NC) and included one general and one under-vaccinated subpopulation for each (i.e. UO, UN, GO, and GN). We used this four-compartment differential equation-based model to explore different assumptions for seasonality and population mixing [3]. As the basis for this analysis, we assumed seasonal fluctuation in transmission consistent with NYS geographic location (i.e. seasonality amplitude of 35%) and explored the effect of three mixing behaviors: (1) *subpopulation isolation* (i.e. the under-vaccinated subpopulations remain isolated from the general ones but mix some between themselves), (2) *no isolation* (i.e. 95% of contacts come from the two subpopulations with the same vaccination levels, while the remaining 5% of contacts come from the other two subpopulations), and (3) *partial isolation* (i.e. 95% of contacts come from each under-vaccinated subpopulation, while the remaining 5% of contacts come from the two general subpopulations) [3].

We applied an established stochastic modeling method to explore the potential for undetected poliovirus transmission in NYS [16–21]. As in the prior studies, we converted the deterministic model into a stochastic model at a fixed point in time. For the present analysis, we started with the results from the deterministic model [3] prior to 1 January 2022, and rounded the fractional number of individuals for all immunity states to the nearest integer based on a random draw. We then used a fixed time step of 0.5 days for the stochastic iteration over the prospective model time horizon and recalculated the rates to update the current state of the model at each time step. After drawing random numbers from Poisson distributions, the model returned the expected number of individuals that transition at each time step, which the model constrained to conserve the total compartment size. For each new infection, we used uniform random draws to determine whether the infection results in a paralytic polio case (if any). In addition, the model estimated the effective proportion of infectious individuals excreting the virus, which wastewater sampling could detect as a signal.

We performed 1,000 distinct realizations of possible futures leading to different potential times when future cases (if any) may occur, which we used as inputs to estimate the confidence about no circulation (CNC) as previously described [16]. For consistency, we used previously described algorithms and specific metrics:

- POE – *P*robability *o*f *E*limination defined as the fraction of stochastic iterations in which die-out occurs
- DEFP – *D*etected-*E*vent-*F*ree *P*eriod defined as the time (*t*) in months since the last detected paralytic case or positive environmental isolate
- CNC – *C*onfidence about *N*o *C*irculation given the DEFP approximated as (1 - the number of DEFPs equal to *t* months with ongoing poliovirus circulation, divided by all DEFPs of *t* months)
- CNCx% – Time when the CNC exceeds x% (i.e. CNC95%)

For the US, we considered the nature of case detection, which occurs in the form of AFM surveillance. This differs from acute flaccid paralysis (AFP) surveillance used in many other regions of the world and in our prior analyses. AFP pathophysiology starts in the lower motor neurons within the spinal cord and continues down the peripheral motor nerve to the innervated muscle and has various causes including paralytic poliomyelitis, transverse myelitis, Guillain–Barré syndrome (GBS), and other infectious and myopathic disorders. The Global Polio Laboratory Network (GPLN) designed AFP surveillance to maximize clinical detection of paralytic polio in children under 15 years of age, with baseline AFP rates used as an indicator for the quality of active surveillance [23,24]. In contrast, AFM represents a more specific diagnostic entity characterized by acute onset of flaccid weakness with radiological evidence of spinal cord gray matter abnormalities on magnetic resonance imaging (MRI) regardless of age [25]. Although the US does not perform routine AFP surveillance, essentially all cases that would meet the surveillance criteria for paralytic poliomyelitis (including those in individuals over 15 years of age) would be identified through clinical and laboratory workups covered under the current AFM surveillance program.

We also considered the nature of the information from the NYS wastewater sampling (WS), which like other high-income countries (e.g. Israel) differs substantially from the environmental surveillance (ES) developed by the GPLN and used in many other regions of the world [22]. WS in the US primarily used convenience samples (e.g. collected for COVID-19 surveillance [26]), analyzed using a pan-poliovirus real-time PCR assay followed by sequencing [2]. Wastewater samples in NYS were processed by ultracentrifugation through a sucrose cushion method [27]. Total nucleic acids were then extracted and tested for polioviruses, and any samples positive for polioviruses were submitted for sequencing [2].

Recognizing the importance of the quality of the information from AFM and WS, we developed detection functions (DFs) similar to prior studies. For AFM surveillance alone, we anticipate that polio cases occur very rarely, and assumed detection of every case (albeit potentially with some delay). Thus, we assumed perfect AFM surveillance, such that *p*_*e*_ = 1 [16,17]. While perfect AFM surveillance may sound sufficient, it may not reveal the presence of transmission in the US because poliovirus transmission can occur in countries with high overall IPV coverage without causing paralysis.

**Figure 1.**
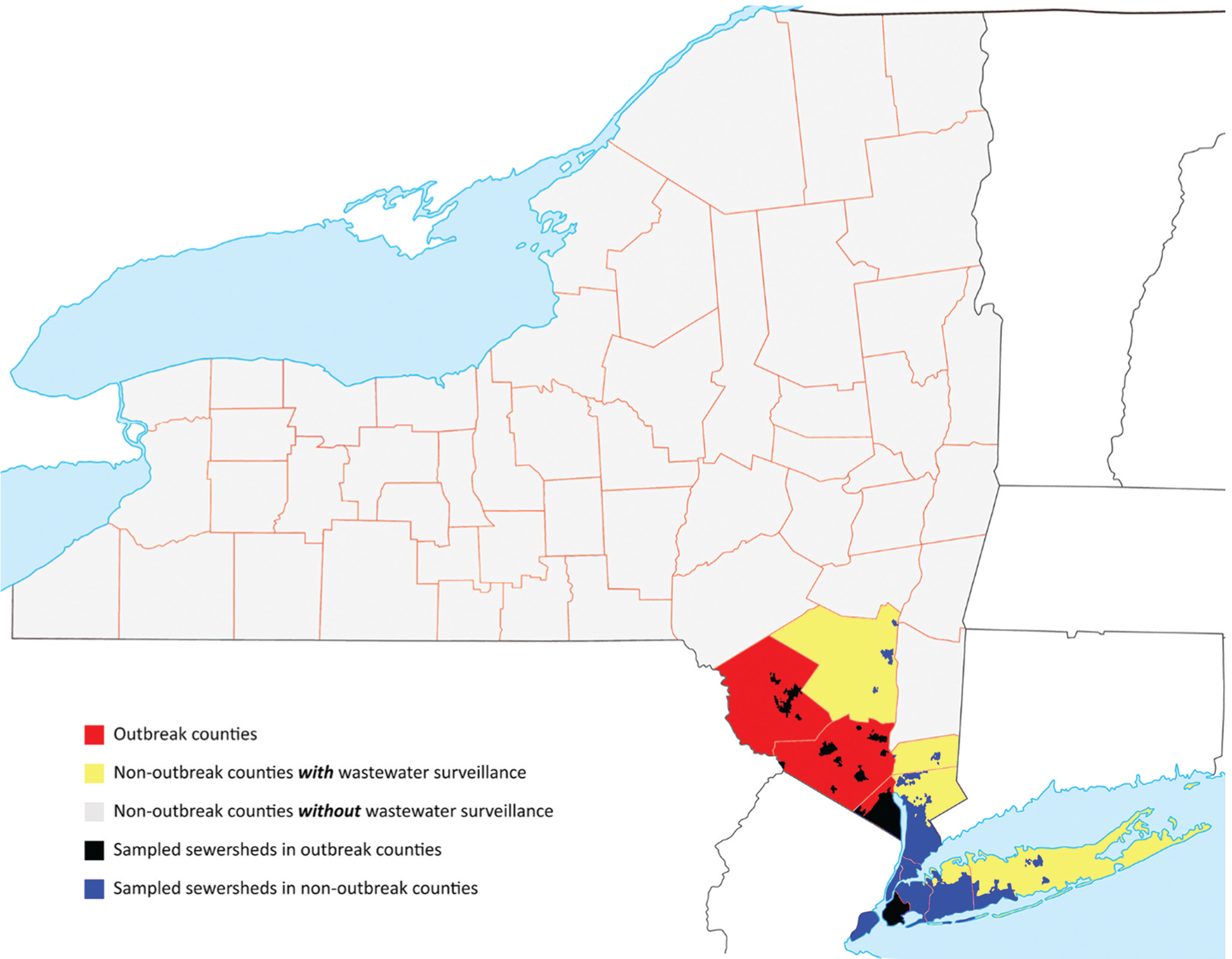
The geographic distribution of wastewater sampling sites for polio with respect to outbreak counties, non-outbreak counties and the NY state in general. Note that sampling sites are primarily in outbreak counties as well as the adjacent non-outbreak counties with high population density.

For WS alone, we defined the DF as the probability of detecting poliovirus in a wastewater sample [16,17]. We used the effective (i.e. infectiousness-weighted) number of infected individuals (*EI*) and a general approach to represent WS in the model, which we referred to as system-wide (SW) in prior studies [16,17]. The SW approach assumes that the DF directly describes the probability of finding poliovirus in any sampling site(*s*) given the fraction of total population covered by all WS sites (*F* > 0) and the prevalence of infected individuals in the population (*EI=N*) [16,17] during weekly sampling according to:

$$s(C)=F^{-C\times \text{In}(EI/N)}$$

where *C* represents a sensitivity coefficient. In this equation, lower values of *C* indicate a more sensitive system, such that 1-*C* scales in the typical definition of sensitivity. Since our modeling approach used more abstract characterization of the different subpopulations [3], and NYS WS catchment areas varied in size and did not conform to county lines [28], we cannot explicitly match the actual WS sampling sites to the modeled subpopulations. Given these challenges, similar to prior modeling, we considered three approaches to distribute WS sites to four subpopulations. First, statewide sites (SWS) assumed the distribution of WS sites equally over the general and under-vaccinated subpopulations. Second, under-vaccinated subpopulation sites (UVS) assumed WS sites represent mostly the under-vaccinated subpopulations, which for the purpose of the results presented here largely correspond to OC. However, we note the very large population of Kings County in the OC reported overall relatively higher immunization coverage, but it includes a relatively small fraction of under-vaccinated communities. Third, general population sites (GPS) assumed that WS sites represent mostly the general subpopulations. Figure 1 shows the geographic distribution of actual WS sites for NYS highlighting the OC and NC with wastewater surveillance, and showing all other NC counties, which we constructed based on publicly available information about the NYS population and sewer-sheds [3,28–30] and poliovirus laboratory data from specific WS sites.

Table 1 shows the estimated fraction of populations under WS using the same data sources as Figure 1, and for which we considered any sewer-sheds with >10 samples tested between March 2022 and October 2023 as under WS for polio. We use the estimates of the fraction of sites in the OC and NC in the fourth column in Table 1 as the best estimates for *F*, and we distribute the WS sites for the three approaches as shown in Table 2. Table 1 also shows the catchment population for each sewer-shed, and fractions of the total population and the population under WS that meet the recommended catchment size of 400,000 for polio surveillance in the US [12]. NYS can (and did) overcome some limitations in sensitivity by extending the duration and/or volume of the sampled wastewater to obtain additional information. Given the relatively new use of WS for poliovirus detection and CNC characterization, we explored the 95% confidence of no circulation (CNC95%) as a function of *C* for the full range from 0 to 1 for the best estimates of the values of *F* from Table 2. We also modeled CNC95% as a function of *C* for the full range from 0 to 1 and *F* from 0.05 to 1 using 0.05 increments for each to develop surface plots that distributed the WS sites without using the information in Tables 1 or 2. Thus, for this sensitivity analysis, we assume less information and do not distribute the WS sites specifically to the OC and NC, but instead GPS only obtains samples from the highly vaccinated general subpopulations, UVS only obtains samples from the under-vaccinated subpopulations, and SWS obtains samples from both.

## 3. Results

**Figure 2.**
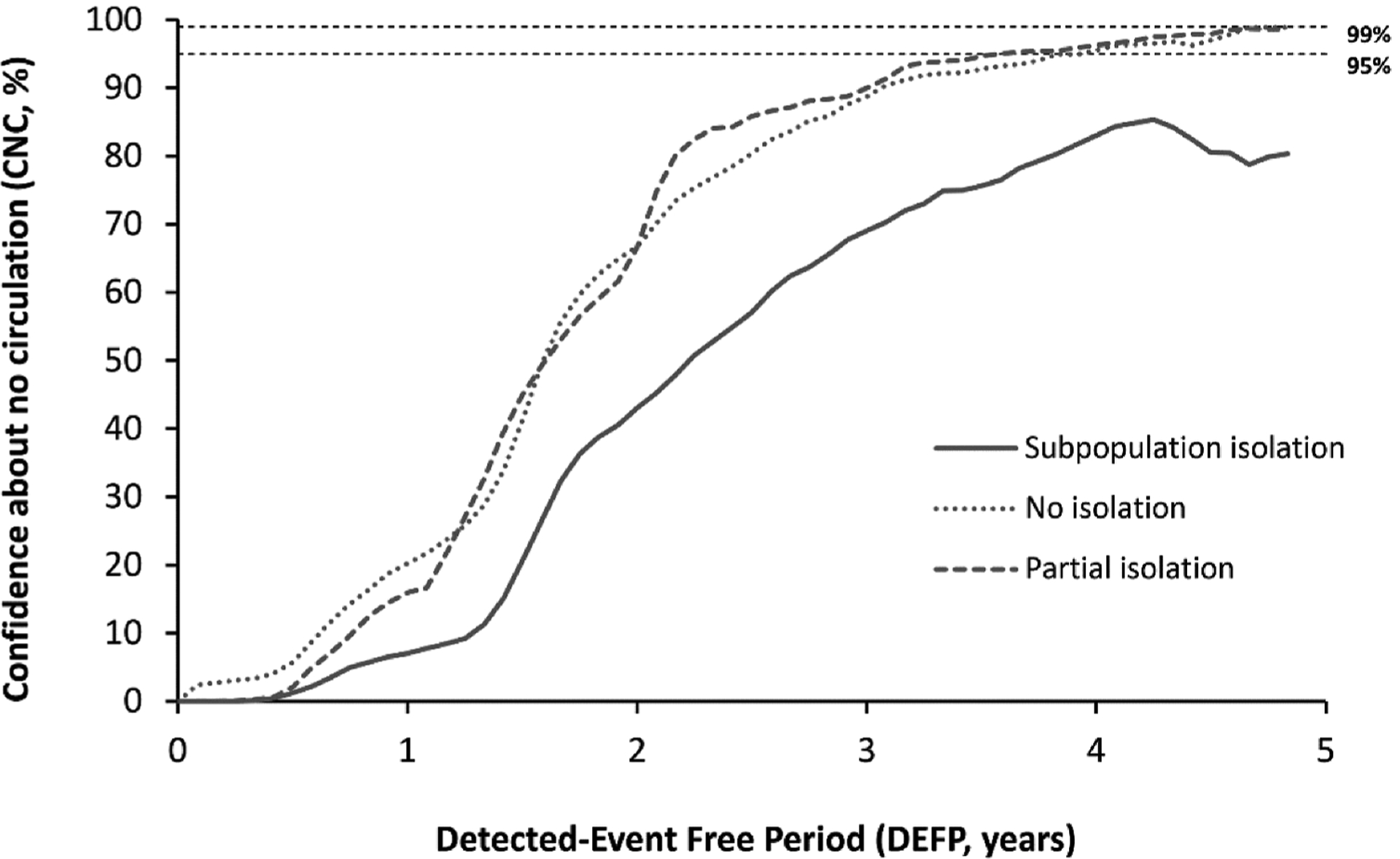
Confidence about no circulation in NYS as a function of the detected-event free period assuming perfect acute flaccid myelitis (AFM) surveillance only (no WS). Dashed horizontal lines indicate 99% and 95% confidence levels for ease of reference. Note that with AFM alone, reaching high confidence of no circulation (95%) it will take approximately 4 years for the *no isolation* and *partial isolation* mixing scenarios, and never occurs for the *subpopulation isolation* mixing scenario for the model time horizon.

Figure 2 shows the CNC95% in NYS as a function of time since the last detected case by perfect AFM only (i.e. no WS). For surveillance with perfect AFM only, depending on the underlying population mixing scenario, the POE ranges from 61% for *Subpopulation isolation* to 77% for *No isolation* to 95% for *Partial isolation*. Notably, for the modeled time horizon of 5 years none of the mixing scenarios reach a CNC99% by AFM only, and only the *No isolation* and *Partial isolation* scenarios reach a CNC95%, which occurs after 4.0 years and 3.7 years from the last detected paralytic case, respectively. These results demonstrate that AFM alone does not provide sufficient information to quickly achieve high confidence as a surveillance strategy. Given the background of high overall IPV immunization rates and relatively low paralysis-to-infection rate for polio, relying solely on AFM surveillance implies needing to wait for many years to reach 95% (CNC95%) and possibly the inability to reach higher confidence.

**Figure 3.**
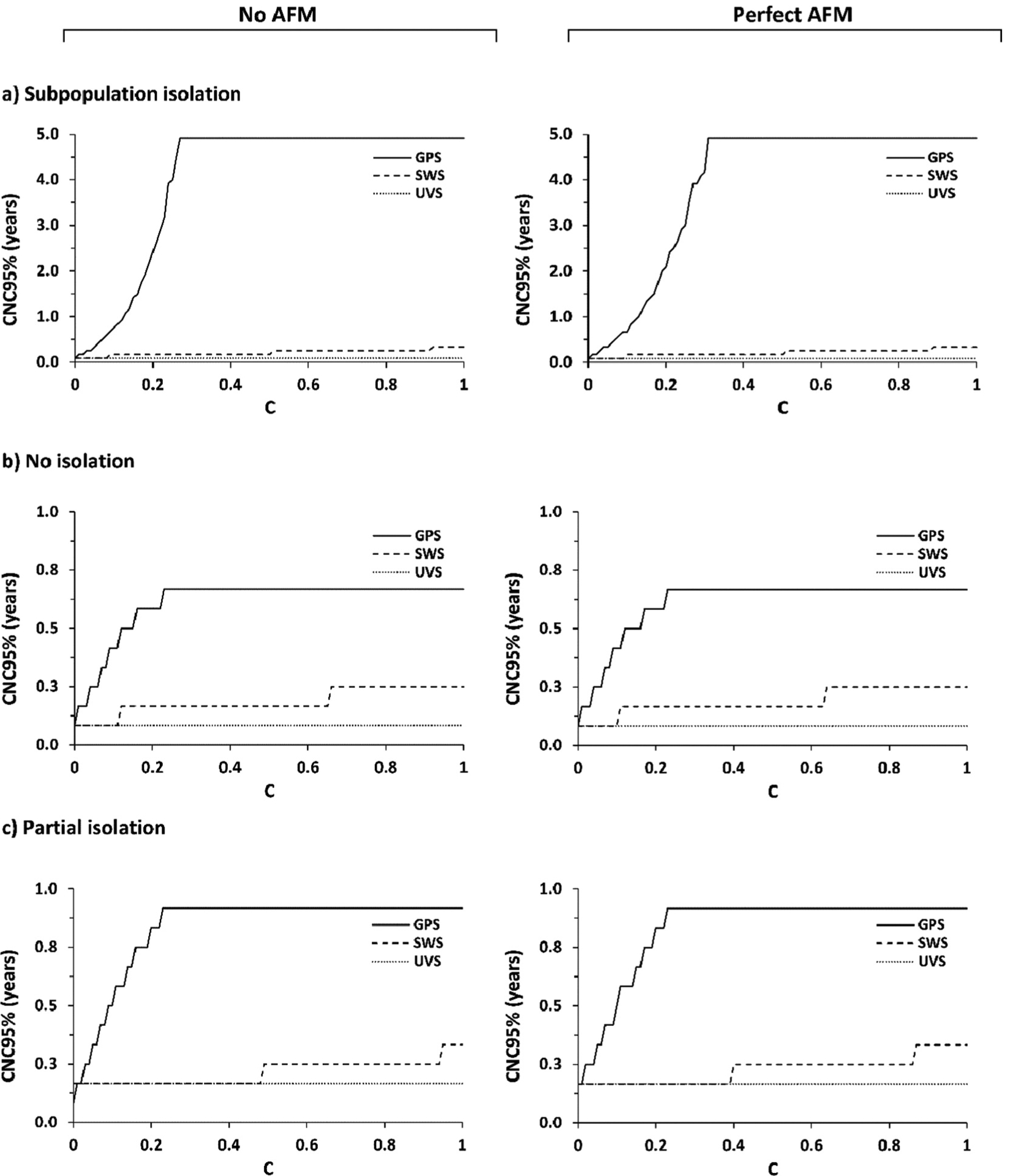
Detected-event-free period in years at which the confidence about no circulation exceeds 95% (CNC95%, z-axis) as a function of the wastewater surveillance (WS) sensitivity coefficient (*C*) assuming no AFM surveillance and perfect AFM surveillance. Results for different mixing assumptions are presented in panels a to c. For each set of results, SWS assumes distribution of WS sites equally over the general and under-vaccinated subpopulations, UVS assumes all WS sites represent only the under-vaccinated subpopulations, and GPS assumes all WS sites represent only the general subpopulations.

Considering the information from WS, Figure 3 shows the CNC95% as a function of the coefficient of sensitivity, *C*. Depending on the amount of poliovirus excreted into the wastewater system, which varies between the population mixing scenarios, and WS location relative to the ongoing transmission (i.e. SWS, UVS, or GPS distribution), good-quality WS can produce substantially shorter estimates of CNC95%. However, Figure 3 shows the CNC95% can range from very short to very long times of ongoing surveillance with no detected events. Notably, in the modeled case in which no transmission enters the general population under the *subpopulation isolation* mixing scenario, the GPS site distribution misses almost all silent transmission and provides limited information relevant to characterizing CNC95% (i.e. *F* = 0.12 for the UOC in Table 2). Figure 3 plateaus for some assumed mixing scenarios and WS site distributions due to the limit of the model time horizon of 5 years, and this censoring means that any value shown at 4.9 years (i.e. 5 years minus 1 month) imply a CNC95% of ≥4.9 years. As shown in Figure 3, the UVS and SWS site distributions capture all or most of the silent transmission and provide good information for characterizing CNC95%. Comparison of the figures on the left with no AFM surveillance to those on the right with perfect AFM surveillance shows the relatively limited information from AFM given the very low expected incidence of cases.

**Figure 4.**
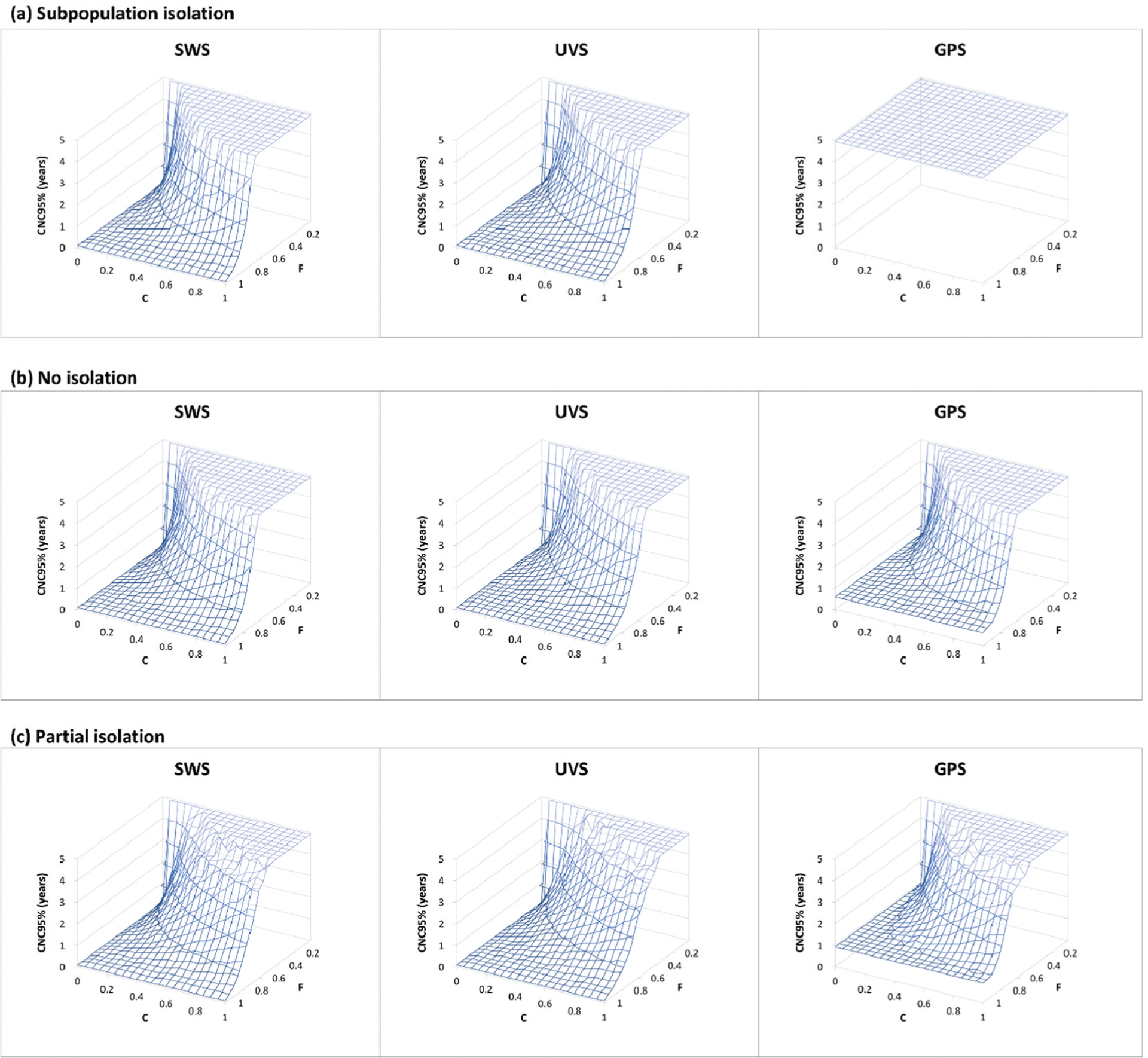
Surface plots of the detected-event-free period in years at which the confidence about no circulation exceeds 95% (CNC95%, z-axis) as a function of the fraction population (*F*) covered by wastewater surveillance (WS) and WS sensitivity coefficient level (*C*) assuming no AFM surveillance (same as figure 3 but zoomed in on the high quality WS range, i.e. high *F* and low *C*). Results for different mixing assumptions are presented in panels a to c. DEFP results are color-coded to highlight different scales of the z-axis. For each set of results, SWS assumes distribution of WS sites equally over the general and under-vaccinated subpopulations, UVS assumes all WS sites represent only the under-vaccinated subpopulations, and GPS assumes all WS sites represent only the general subpopulations.

The results presented in Figures 2–4 highlight some of the uncertainty related to the interpretation of the information shown in Figure 1 and Table 1 with respect to the actual WS performed in response to the 2022–23 NYS outbreak. The data presented in Table 1 and Figure 1 show that the NYS WS primarily focused on OC and the surrounding parts of NC, with relatively high WS coverage in the most populated parts of the at-risk communities. Table 1 also shows that WS in both Sullivan and Orange counties covered less than half of the populations in those counties, with large numbers of individuals on septic systems, and the population in Sullivan potentially large enough to sustain transmission depending on the immunization coverage and mixing. In addition, Table 1 shows the large catchment size of WS in some densely populated urban centers, which may have limited the wastewater detection sensitivity given the very limited scope of this outbreak.

Figure 4 provides a sensitivity analysis by showing the full surface for the CNC95% as a function of both *F* and *C*. Notably, if WS only covers the highly immunized general population, then sampling only the GPS shows censored results (i.e. uninformative WS) for the *subpopulation isolation* mixing scenario for high values of *C* (compare GPS site distribution results in panel a of Figures 3 and 4) that reach CNC95% of ≥4.9 years. The results in Figure 4 show that for all mixing scenarios and possible site distribution options, some values of *F* and *C* (i.e. low *F* and high *C*) include this censored area, which highlights the importance of selection and quality of WS sites.

## 4. Discussion

The analysis suggests that with ongoing, active, and high-quality WS, public health officials could assume 95% confidence of no ongoing circulation of the outbreak poliovirus within approximately 3–10 months since the last positive wastewater sample or polio case (whichever occurs later). With the last reported NYS detection in February 2023, if the WS is highly sensitive (i.e. low *C*), this could imply a high CNC as early as June 2023. However, continued surveillance is required to achieve very high CNC for a broader range of *C* for all different approaches. Considering the available information about the NYS WS and uncertainty about the sensitivity of the system, achieving sufficiently high CNC may require longer times with active and ongoing surveillance. During that time, any signal detected requires thorough investigation. Any positive detection of the outbreak poliovirus restarts the clock for the DEFP (e.g. the December 2022 detection reset the clock 6 weeks after the last prior detection, the February 2023 detection reset the clock again 10 weeks later, and as of October 2023, the DEFP now exceeds 28 weeks).

Efforts to reduce uncertainty about the actual quality of WS could include more detailed analyses of the sensitivity and specificity of the NYS WS system with respect to detecting rare poliovirus events. This may include the development and testing of methods for overcoming the complexity of sampling large urban population centers for circulating polioviruses, experiments that evaluate the sensitivity of the collection and/or assay methods, and engagement of WS and/or community experts. Although environmental surveillance for polioviruses dates back several decades, the current WS systems in NYS used a platform primarily designed to address public health issues related to COVID-19, and as such relies on different laboratory methods than those used historically for poliovirus environmental surveillance [22]. Further studies are needed to compare this method to traditional and internationally standardized methods and to characterize the sensitivity of WS in NYS.

The NYS outbreak experience suggests that WS should continue for a sufficiently long enough period for public health authorities to obtain high CNC. When WS stops, the CNC curve will reflect the absence of WS information and revert at that point to the confidence obtained from AFM alone, as well as any censoring associated with the model time horizon. These results highlight the importance of selecting WS site locations and scope relative to populations at high risk of transmission. Looking for polioviruses in the well-vaccinated general population alone will not likely provide helpful information. The NYS WS for polio prioritized sampling in and around the outbreak counties, which should serve as a model for any potential future outbreaks in polio-free countries.

WS itself comes with other challenges, including financial costs, requirements for laboratory and human resources, and the reality that public health authorities may need to manage issues that arise with communication of single detections without evidence of local transmission [7] and/or interpretation of false positives and/or weak signals, which are unavoidable in any laboratory testing system. The detection of polioviruses in wastewater typically involves PCR amplification of targeted sequences to identify potential signals for all polioviruses, with a high cycle threshold (C_t_), indicating a very low signal and creating interpretation difficulties [31]. In the absence of more definitive signals, repeated high C_t_ PCR results could be interpreted as (i) assay false positives, (ii) ongoing transmission of a specific outbreak virus that is below the assay sensitivity level, or (iii) a transient excretion from a poliovirus-infected traveler (including an OPV recipient) into the local sewer system that does not reflect local transmission. Public health authorities who observe high C_t_ signals may want to consider increasing sample collection volume, duration, and/or frequency to increase sensitivity during peak seasons or changes in population movements. The integrative nature of WS also implies that public health authorities face challenges with identifying whether detections indicate local transmission or not, because the specific source of the poliovirus signal does not directly connect to one or more identified individuals.

The under-vaccinated populations in NYS and elsewhere in the US pose continued vulnerability of these populations to imported polioviruses [9]. Public health authorities are expected to continue efforts to immunize under-vaccinated populations and should recognize the benefits of protection from all three types of polioviruses provided by IPV. IPV delivery in the NYS emergency response for this outbreak offered protection for previously unvaccinated individuals who received it, and most likely played a relatively minor, but positive, role in reducing the outbreak transmission [3]. The use of IPV to respond to this outbreak contributes to the growing evidence base of IPV use for outbreak response, which includes mixed experience to date [32]. Notably, some IPV-only using countries historically used oral poliovirus vaccine (OPV) to respond to outbreaks [33]. In 2013–2014, IPV use in Israel did not stop transmission of an imported type 1 wild poliovirus, and this led to the use of bivalent OPV (bOPV) to stop the outbreak and the reintroduction of bOPV into routine immunization [10,34].

More recently, following the globally coordinated cessation of type 2 OPV use in routine immunization (OPV2 cessation), all countries became IPV-only using countries with respect to type 2 polioviruses. Since OPV2 cessation, China has successfully responded to type 2 outbreaks with IPV [35]. In contrast, the use of IPV in Tajikistan did not stop transmission, which led to the use of OPV [36]. In 2022, the UK, the US, and Israel used IPV to respond to cVDPV2 transmission, with the last detections to date reported November 2022 in the UK [37] and February 2023 in the US [3] and Israel [38]. In general, the ability of IPV to stop transmission of a poliovirus outbreak will depend on the level of population immunity, transmission potential of outbreak poliovirus, impact of seasonality, and mixing dynamics. Further modeling should explore the characteristics of outbreaks for which IPV use may stop the outbreak and those that may require the use of OPV to stop transmission.

Similar to our prior modeling studies that explored the potential for undetected transmission of polioviruses [13–18], this analysis comes with several limitations. First, the use of a transmission threshold as the die-out criterion, rather than absolute 0 total infected individuals, represents a simplified construct of the complex dynamics of transmission die-out. Second, the nature, quality, and uncertainties related to WS influence our assumptions about WS-related methods and model inputs, which in turn lead to a wide range of results. Third, while we chose a simplified approach to model WS (given the level of abstraction of the model), its actual ability to detect poliovirus depends on the choice of sampling site, the size and composition of the catchment population, the frequency of collection, and the daily viral yield, the methods for sample collection and virus concentration, and the ability to recover and identify poliovirus from concentrated WS sample. Despite its limitations, we believe the analysis provides useful insights by exploring a range of possibilities and some of the differences between poliovirus surveillance in high-income IPV-only using countries like the US (i.e. AFM and WS) and other countries modeled previously [16–21].

## 5. Conclusions

The poliovirus responsible for the NYS outbreak most likely lost steam during the low season, with decreasing detections in the winter and the last detection in February 2023. Since WS in NYS focused on the highest risk areas and provided good coverage for an extended period of time, this modeling supports high confidence that the outbreak ended, consistent with the decline of positive signals in WS in NYS. Additional surveillance will continue to provide confidence about the die out of the transmission of this outbreak virus and may detect future signals.

## Figures and Tables

**Table 1.** New York State Wastewater characteristics for polio by outbreak and non-outbreak counties.

	Total population*	Population under WS for polio	Fraction of population under WS for polio**	Catchment population meeting recommended limit***	Fraction of total population meeting recommended limit***	Fraction of population under WS meeting recommended limit***
**Outbreak Counties**	**3,415,137**	**3,109,375**	**0.91**	**1,065,801**	**0.31**	**0.34**
Kings	2,590,516	2,590,516	1	546,942	0.21	0.21
Orange	405,941	163,725	0.40	163,725	0.40	1.00
Rockland	339,022	339,022	1	339,022	1	1
Sullivan	79,658	16,112	0.20	16,112	0.20	1.00
**Non-outbreak Counties**	**16,262,014**	**8,067,586**	**0.50**	**1,322,239**	**0.08**	**0.16**
Bronx	1,379,946	1,379,946	1	0	0	0.00
Nassau	1,383,726	1,180,961	0.85	50,481	0.04	0.04
New York	1,596,273	1,596,273	1	0	0	0
Putnam	98,045	4,480	0.05	4,480	0.05	1.00
Queens	2,278,029	2,246,137	1	131,254	0.06	0.06
Richmond	491,133	491,133	1	491,133	1	1.00
Suffolk	1,525,465	292,063	0.19	292,063	0.19	1.00
Ulster	182,319	37,176	0.20	37,176	0.20	1.00
Westchester	990,427	839,417	0.85	315,652	0.32	0.38
All other	6,336,651	0	0	0	0	0
**Total NYS**	**19,677,151**	**11,176,961**	**0.57**	**2,388,040**	**0.12**	**0.21**

* Total population data based on US Census Bureau estimates for 2022 [30].

** For Kings, Rockland, Bronx, New York, Queens, and Richmond counties, fraction of population under WS is set to 1 given that these counties are nearly entirely under public sewer system [28]. For all other counties, the fraction is calculated based on population under WS divided by the total population.

*** Recommended catchment area limit of 400,000 people [12].

**Table 2.** Assignment of the percentage of New York State poliovirus WS observations for each mixing scenario for the.

	Model subpopulations				Separation of outbreak counties		State total
	UO	GO	UN	GN	OC	NC	NYS
% NYS population	1.8%	15.8%	1.2%	81.2%	17.6%	82.4%	100.0%
Statewide sites (SWS)	0.91	0.91	0.50	0.50	0.91	0.50	0.57
Under-vaccinated subpopulation sites (UVS)	1.00	0.90	1.00	0.49	0.91	0.50	0.57
General populations sites (GPS)	0.12	1.00	0.00	0.51	0.91	0.50	0.57
